# A study of the validity and reliability of the questionnaire entitled “physicians' approach to and disclosure of medical errors and the related ethical issues”

**Published:** 2019-03-06

**Authors:** Mohammad Mohammadi, Bagher Larijani, Seyed Mahmoud Tabatabaei, Saharnaz Nedjat, Masud Yunesian, Fatemeh Sadat Nayeri

**Affiliations:** 1 *PhD Candidate in Medical Ethics, Medical Ethics and History of Medicine Research Center, Tehran University of Medical Sciences, Tehran, Iran; Department of Medical Ethics, School of Medicine, Tehran University of Medical Sciences, Tehran,* *Iran; Faculty of Medicine, Department of Medical Ethics, Mashhad University of Medical Sciences, Mashhad, Iran.*; 2 *Professor, Endocrinology and Metabolism Research Center, Endocrinology and Metabolism Clinical Sciences Institute, Tehran University of Medical Sciences, Tehran, Iran* *.*; 3 *Professor, Medical Ethics and History of Medicine Research Center, Tehran University of Medical Sciences, Tehran, Iran.*; 4 *Professor, Knowledge Utilization Research Center, Tehran University of Medical Sciences, Tehran, Iran; Department of Epidemiology and Biostatistics, School of Public Health, Tehran University of Medical Sciences, Tehran, Iran.*; 5 *Professor, Department of Research Methodology and Data Analysis, Institute for Environmental Research, Tehran University of Medical Sciences, Tehran, Iran; Department of Environmental Heath, School of Public Health, Tehran University of Medical Sciences, Tehran, Iran.*; 6 *Professor, Maternal-Fetal and Neonatal Research Center, Tehran University of Medical Sciences, Tehran, Iran.*

**Keywords:** Medical errors, Disclosure, Beneficence, Non-maleficence, Personal autonomy

## Abstract

Medical errors are among the major challenges that threaten patients’ health worldwide. The aim of this study was to design a valid and reliable questionnaire to investigate the status of medical error disclosure by physicians.

A preliminary questionnaire was developed based on the extracted results from 37 interviews with specialists. To test the validity of the questionnaire, 20 medical practitioners and medical ethics authorities were asked to evaluate the relevance and clarity of each item. To measure the instrument’s reliability (the intra-class correlation coefficient and Cronbach’s alpha), a test-retest study was conducted on 20 randomly selected physicians twice with a 2-week interval. Statistical analyses were performed using SPSS software version 20.

The overall relevance and clarity of the instrument, with an average approach, were measured at 97.22 and 94.03 percent respectively. The Cronbach’s alpha, which presents the internal consistency was satisfactory (0.70 - 0.79) for various domains of the questionnaire. The range of intra-class correlation coefficients for the items in all domains of the questionnaire was 0.76 to 1.00.

Regarding the validity and reliability of the questionnaire, it can be an appropriate instrument in the assessment and monitoring of the status of medical error disclosure by physicians.

## Introduction

Error is an integral part of human life (1), and medical errors are among the major challenges to patients’ health worldwide (2). Regardless of how skilled, committed, or careful they are, people commit errors, and healthcare team members are no exception (3, 4). The occurrence of medical errors is inevitable due to factors such as the complexity of medical knowledge, time constraints, and the need for action despite inadequate and uncertain information (5).

A medical error has been defined as an act of omission or commission, which is either a failure to fully implement the planned measures or to use of a wrong method to achieve a goal, with or without any harm to the patient (6). Medical errors are also regarded as unintentional and unexpected but preventable adverse events in medical care (7 - 9). Based on some classifications, there are three types of medical errors: serious, minor and near-miss. A serious or major error is one that gives rise to permanent or transient injuries that may be life-threatening, whereas a minor error brings about harms that are neither permanent nor potentially life-threatening. Finally, a near-miss error is one that could have inflicted harm but did not, as a result of timely intervention or through sheer luck (10). 

Recent studies have indicated an increase in the rank of mortality due to medical errors in the United States. It is estimated that more than 250,000 deaths every year are due to medical error, which has risen in rank from the eighth cause of death in 1999 to the third in 2013 (11, 12). 

Empirical studies have shown that patients tend to be aware of medical errors, the causes and associated complications, and preventive measures to avoid their repetition (13). Medical error disclosure actually falls under the category of respect for patient autonomy and health, and provides the possibility to obtain informed consent from the patient for the treatment of error-induced damages (6). From an ethical point of view, patients have the right to receive information about diagnostic or therapeutic measures before their implementation, and as a result are entitled to know the consequences of such measures (14). Moreover, in cases of medical error, patients’ healthcare takes priority over commitment to organization (15). Several studies have demonstrated that the majority of patients prefer to be informed when errors are made, but physicians are mostly reluctant to disclose errors and tend to provide no or inadequate information (16 - 19).

With regard to physicians’ commitment on error occurrence, opinion 8.12 of the code of medical ethics of the American Medical Association asserts physicians to “disclose medical errors if they have occurred in the patient’s care, in keeping with ethics guidance” (20).

Error disclosure reflects physicians’ honesty and truthfulness, which can reduce patients’ discomfort, cultivate trust and confidence, and evoke their positive emotional response; it also fulfills their need to get informed about the quality and manner of their care and promotes awareness of their current status, which will in turn facilitate obtaining informed consent for the treatment of error-induced damages and a peaceful compensation (6, 21).

It is difficult to get an overall picture of medical errors in developing countries (22), which may be due to absence of a proper recording and reporting system and limited research studies in this area (23). 

There have been few studies about the attitudes of physicians toward medical errors and the proper manner to deal with them. Studies on the subject are rather difficult to conduct due to ethical issues and an absence of a valid and reliable questionnaire to assess physicians’ approach to and disclosure of medical errors. Although many studies have been conducted on medical errors in Iran, this is the first one, to our knowledge, that attempted to deal with the construction and validation of a questionnaire on medical error disclosure.

This study aimed to design a valid and reliable questionnaire to investigate the practices of Iranian physicians and their colleagues in dealing with medical errors, especially disclosure and the manner in which it is done, as well as the related ethical issues.

## Methods

This study was conducted in two phases. In the first phase, the questionnaire was designed, and in the second, its reliability and validity were measured.

***Phase I: Questionnaire Design***

Initially, a semi-structured interview was performed with 37 medical specialists about medical error disclosure. The main questions of the interview explored the attitudes, concerns, experiences and practices in dealing with medical errors (either by the physicians themselves or their colleagues), as well as medical error disclosure, recording and reporting, and legal issues. The number of interviews were calculated to be 24 based on the opportunistic maximum variation sampling approach. The variables included gender (male and female), age (less than 45 years and more than 45 years), duration of medical practice (less than 20 years and more than 20 years), and type of specialization (internal medicine, surgery, and other specialties). Data saturation was achieved with 37 interviews (Table 1).

**Table 1:** The characteristics of interviewees

Variables	Variable Characteristics	N
Type of Specialization	Internal medicine	15
	Surgery	14
	Other specialties (four pediatricians, one psychologist, one radiologist, and two forensic medicine practitioners)	8
Gender	Male	11
	Female	26
The mean period of practice (years)	21.25 ± 10.27	

In the first round, open coding was conducted and 93 codes were obtained, which were assigned into four categories. Then, during axial coding, the characteristics and the ranges of their values were presented in subcategories (Table 2). 

**Table 2:** The development of categories and subcategories after interview analysis

Category 1: The nature and theoretical foundations of medical error	
Subcategories	Definition and types of medical errors Consequences of medical errors Ethical/philosophical foundations Legal issues
Category 2: Medical error management	
Subcategories	Medical error disclosure Obstacles to medical error disclosure Apology Compensation for medical errors Responsibility Systematic approach
Category 3: Colleagues’ medical error management	
Subcategories	Informing colleagues Colleagues’ medical error disclosure to patients Dealing with colleagues’ errors
Category 4: Documentation of medical errors	
Subcategories	Medical error recording (on patient medical records) Error reporting (to the health system)

As the next step, two researchers familiar with the notions of medical errors and medical ethics extracted factors, components and concepts related to medical errors from the categories and subcategories (including characteristics and the ranges of their values). At the end of this round, 48 items were obtained based on which 48 questions were designed. 

In the second round, based on the aims of the study, the preliminary questionnaire was revised using the comments of a panel of experts familiar with questionnaire design. The panel consisted of two medical ethicists, two medical specialists with academic and managerial background of Iranian medical ethics centers, and one epidemiologist. 

The most important aspect of the questionnaire content was physicians’ attitudes toward medical error disclosure by themselves and their colleagues, and the manner of disclosure. Other items such as physicians’ practices in dealing with errors and their recording and reporting were also included, as documentation and a kind of medical error disclosure to the organization. Moreover, three categories of medical errors were identified: serious (major), no harm (minor), and near-miss. Finally, after the review, deletion, merging, and summarizing of previous items and questions, 36 new items were obtained, based on which the preliminary version of the questionnaire was designed.

***Phase II: Reliability and Validity of the Questionnaire***

*A. Content Validity (Relevance and Clarity)*

In the next round, we investigated relevance, clarity of questionnaire items, and comprehensiveness of the instrument in order to measure content validity. For external validity, the items were evaluated in terms of appropriateness for the target population (including 20 general practitioners and medical specialists), considering subject assessment, format and appearance of the questionnaire. A four-point Likert scale with responses ranging from undesirable, somewhat desirable, desirable, and very desirable was used to evaluate the relevance and clarity of each item separately. 

Item content validity index (I-CVI) and scale content validity index (S-CVI) were used to analyze the participants’ views in terms of the relevance and clarity of each item or the scale, respectively. The I-CVI was calculated by the proportion of participants who had chosen the responses “desirable” or “very desirable” to the total number of participants, and the S-CVI was the mean of the I-CVIs for all items on the scale (24 - 27). 

The instrument was also assessed for comprehensiveness. Corrective remarks were discussed individually with the participants, and constructive comments were taken into consideration. Subsequently, the questionnaire was revised and necessary amendments and modifications were made while retaining the original content.

*B. Reliability*

To measure the instrument’s reliability, a test-retest was conducted with 20 randomly selected physicians twice with a 2-week interval. Internal consistency was determined using the Cronbach’s alpha, and for the test-retest, the intra-class correlation coefficient (ICC) was calculated. At this stage, the items in each domain were examined, and the responses to some items were reversed so they would be aligned in each domain. Cronbach’s alpha coefficients were obtained for each item, and in order to estimate intra-class correlation, the total score for each domain was evaluated. Ten items that were in line with the objectives of the research were not categorized in any of the domains because they were presented as single questions and were not related to other items in different sections of the questionnaire. Cronbach’s alpha was therefore not obtained for such items, and only the reliability coefficient based on intra-class cluster correlation was calculated. SPSS software version 20 was used for data analyses. 

The final version of the questionnaire was constructed in three parts. The first part provided definitions of medical error, its types, and issues pertaining to medical error disclosure, the second part included items related to the three domains of medical error, and the third presented demographic information.

***Ethical Considerations***

This study and the accompanying questionnaire were approved by the ethics committee of Tehran University of Medical Sciences before they were completed by the physicians in the test-retest step (IR.TUMS.VCR.REC.1395.1535). Participation in the study was entirely voluntary.

## Results

The final version of the questionnaire in the second part comprised 36 questions categorized in three domains (Table 3).

**Table 3:** Domains of items in the medical error disclosure questionnaire

Domain	Number of Itemsɰ	Number of Items in Subdomainɸ
**Physicians’ practices in dealing with medical ** **errors **	9	22
**Disclosure** **1. Medical error disclosure (either it was ** **committed by the physician or other colleagues)** **2. Medical error recording and reporting**	7 2	11 2
**Manner of medical error disclosure**	8	8
**Items with no categorization**	10	10

ɰ *Number of questions or items in each domain. Some questions or items investigated two or three parameters (subdomains). For example, the physicians were asked,* *“* *Have you committed a medical error within the past 6 months?” about three types of errors, that is, serious (major), no harm (minor) and near-miss* *.*

ɸ *The sum of these parameters (subdomains) is presented in the second column.*

Given that in the questionnaire, medical errors were investigated from several aspects, each domain was separately evaluated in the validity and reliability tests.

In the content validity phase, the participants consisted of 10 ethicists (all physicians) and 10 specialists. The results of the study showed that the relevancy and clarity of each item (I-CVI) was at an acceptable level (above 80%), as can be seen in Table 4. The overall relevancy and clarity of the instrument, with an average approach, were measured to be 97.22 and 94.03 percent, respectively (S-CVI). The comprehensiveness of the instrument was also excellent (100%). It should be added that three questions had to be improved in terms of language with content preservation.

**Table 4:** The relevancy and clarity of each item in the medical error disclosure questionnaire

Item No.	Relevancy		Clarity	
	*The number of observed agreements*	*I-CVI*	*The number of observed agreements*	***I-CVI***
**1**	19	95	19	95
**2**	18	90	19	95
**3**	20	100	19	95
**4**	20	100	19	95
**5**	20	100	20	100
**6**	19	95	20	100
**7**	19	95	19	95
**8**	19	95	16	80
**9**	20	100	19	95
**10**	18	90	18	90
**11**	19	95	19	95
**12**	20	100	19	95
**13**	20	100	19	95
**14**	19	95	19	95
**15**	19	95	18	90
**16**	20	100	18	90
**17**	19	95	18	90
**18**	19	95	19	95
**19**	20	100	17	85
**20**	19	95	19	95
**21**	20	100	20	100
**22**	19	95	19	95
**23**	20	100	20	100
**24**	20	100	20	100
**25**	19	95	19	95
**26**	20	100	20	100
**27**	20	100	20	100
**28**	19	95	19	95
**29**	18	90	17	85
**30**	19	95	17	85
**31**	20	100	19	95
**32**	20	100	19	95
**33**	20	100	19	95
**34**	20	100	18	90
**35**	20	100	19	95
**36**	20	100	19	95

In the test-retest phase, participants included 12 men and 8 women (9 general practitioners and 11 specialists). Participants’ ages ranged between 35 and 60 (44.85 ± 8.25) years, and their mean period of practice was 15.35 ± 8.86 years.

The results were satisfactory in all domains of the questionnaire (Cronbach’s alpha: 0.70 - 0.79). The range of ICC for the items in the domain of “physicians’ practices in dealing with medical errorˮ was 0.76 to 1.00, and that of “medical error disclosure (either it was committed by the physician or other colleagues)” was 0.79 to 0.98. ICC ranged between 0.87 and 0.95 for the items in the domain of “medical error recording and reporting” and between 0.87 and 0.97 for the items in the domain of “Manner of medical error disclosure”.

**Table 5:** Cronbach’s alpha coefficients and test-retest reliability of items in three domains of the medical error disclosure questionnaire

Domain	Cronbach's Alpha	ICC¶	ICC Range
**Physicians’ practices in dealing with medical errors **	0.76	*	0.76 - 1.00
**Disclosure** **1. Medical error disclosure (either it was ** **committed by the physician or other colleagues)**	0.74	0.95	0.79 - 0.98
**2. Medical error recording and reporting**	0.79	0.92	0.87- 0.95
**Manner of medical error disclosure**	0.70	0.72	0.87 - 0.97
**Items with no categorization**	-	*	0.90 - 1.00

¶ *Intra-class correlation coefficient*

* Since the ICC could not be calculated in the domain of practice due to the lack of scores for each item and, consequently, there was no total score, the ICC of each item was separately calculated twice (test-retest), in which correlation of all items exceeded 0.7. Pearson's correlation was performed for each item and was higher than 0.7.

Table 5 presents the values of Cronbach’s alpha coefficients and test-retest reliability in each domain of the questionnaire. The values of Cronbach’s alpha coefficients and the test-retest reliability of each item are demonstrated in Table 6. At this point, the appropriateness of the final version of the questionnaire was confirmed considering the acceptable results of the statistical tests.

**Table 6:** The ICC¶ and Cronbach’s alpha of each item in the final version of the questionnaire

**#**	Items	ICC of Each Item	Cronbach's Alpha if Item is Deleted
**1**	Have you committed a medical error within the past 6 months?	0.92	0.77
**2**	Have you noticed a medical error by your colleagues over the past six months?	0.99	0.71
**3**	Have you ever disclosed a major medical error to your patients?	0.96	0.74
**4**	Have you ever disclosed a minor medical error (no harmful event) to your patients?	0.98	0.78
**5**	Have you ever apologized for your medical error?	1.00	0.78
**6**	Have you ever been sued for a medical error by a patient who has been notified of the error through sources other than yourself?	1.00	0.75
**7**	Have you ever been sued for a medical error by a patient after you informed him/her of the error?	0.97	0.72
**8**	Have you ever recorded your medical error?	0.93	0.69
**9**	Have you ever reported your medical error to your organization?	0.76	0.65
**10**	What percentage of patients do you think may complain about their physicians when they are informed of a medical error through sources other than their physicians?	0.99	*
**11**	What percentage of patients do you think may complain about their physicians after error disclosure by the physicians?	0.99	*
**12**	To whom should a medical error be disclosed?	0.99	1.00 ɰ,*
**13**	Who should disclose the medical error to the patient?	1.00	1.00 ɰ,*
**14**	In general, it is ethically necessary to disclose a medical error to a patient?	0.95	0.34
**15**	In general, it is ethically correct to disclose a colleague’s medical error to his/her patient?	0.95	0.75
**16**	In general, it is ethically correct to document a medical error (committed by yourself) in the patient’s medical record?	0.94	0.61
**17**	In general, it is ethically correct to report a medical error (committed by yourself) to your organization?	0.87	0.67
**18**	In any circumstance, the physician must inform the patient of his/her medical error.	0.79	0.85
**19**	Talking honestly and frankly about an error that I committed is acceptable in my workplace.	0.92	*
**20**	Error non-disclosure must be reasonably justified and approved by an impartial individual or group (for instance the ethics committee).	0.95	*
**21**	If the patient does not become aware of a medical error, it is not necessary to disclose it to him/her.	0.96	0.58
**22**	It is not mandatory for the physician to disclose the error until the patient inquires about it.	0.92	0.60
**23**	Error disclosure to the patient should be made sincerely, honestly, and with a regretful expression.	0.88	0.64
**24**	Error disclosure should be made with an explanation of this preventable event, error-induced damage, prognosis and possible treatments.	0.95	0.67
**25**	The physician should apologize to the patient and assure him/her that the same error will not occur to another patient in the future.	0.97	0.76
**26**	Details of medical error disclosure to the patient must be documented.	0.87	0.68
**27**	It is not necessary to disclose the error to the patient, but his/her treatment should be carried out/continued by the physician until complete resolution of the error- induced complications.	0.92	0.66
**28**	It is not necessary to disclose the error to the patient, but the physician must bear the costs of treatment for the medical error.	0.97	0.72
**29**	Until the judiciary condemns the physician to pay compensation (Diyah) for the damages, such costs should not be paid by the physician.	0.94	*
**30**	If a colleague commits a medical error, we should ask him/her to inform the patient.	0.92	0.68
**31**	If our colleague fails to disclose his/her error to the patient and we are aware of it, we should disclose the major medical error to the patient.	0.91	0.63
**32**	Avoiding colleagues’ error disclosure might jeopardize the medical profession and dignity of the medical community.	0.97	0.66
**33**	Physicians committing errors repeatedly should be referred to competent authorities by their peers to resolve the issue.	0.96	0.74
**34**	Medical errors and the underlying reasons should be discussed with peers in order to devise strategies to minimize their occurrence	0.83	*
**35**	Medical errors and the underlying reasons should be analyzed by managers and physicians in order to devise strategies for their reduction.	0.88	*
**36**	Identify three main reasons for medical error non- disclosure to the patient.	0.9 - 1.00	*

¶ *Intra-class correlation coefficient*

* *Items with no categorization*

ɰ *Kappa coefficient was calculated for these items.*

## Discussion

The need for a valid and reliable questionnaire to explore the practices of Iranian physicians and their colleagues in dealing with medical errors, especially disclosure and the manner in which it is conducted, as well as the related ethical issues urged us to conduct the current study. 

The existing questionnaires have only addressed some aspects of medical error and its disclosure. In a study conducted in Turkey on 652 members of a general hospital medical staff during 2010, Cronbach’s alpha coefficient for the participants’ attitude toward medical errors was 0.66 (28). They had used a questionnaire with 16 items in three domains of ‘perception of medical errors’, ‘approach to errors’, and ‘reasons for errors’. Over 50% of the questions overlap with those in our study, although we also explored their history regarding committing medical errors, error disclosure, recording and reporting, as well as physicians’ attitudes toward medical errors, manner of disclosure, and physicians’ approach to their colleagues’ errors. Moreover, our instrument showed a higher validity and reliability compared with their study (28).

In another study by Kim et al., healthcare professionals’ attitudes toward teamwork and safety in South Korea were explored. A part of their questionnaire deals with medical errors, and there are two sections entitled ‘error/procedural compliance’ and ‘error management’ consisting of 11 items. These items examine participants’ attitude toward the nature of medical errors, the causes, the approach of institutions and other physicians to medical errors, committing medical errors, and prevention of their recurrence. In comparison with our more comprehensive questionnaire, they only used a small number of questions in a limited number of domains, and the Cronbach’s alpha coefficients for ‘error/procedural compliance’ and ‘error management’ were 0.212 and 0.156, respectively (29).

In their study on 831 physicians in 2002, Blendon et al. used a 29-item questionnaire that addressed issues such as medical error experience (errors made in their own or their family members’ care), the frequency and causes of medical errors, and the effectiveness of possible error-reduction strategies. Some of their questions overlap with those of our questionnaire, but they did not address the physicians’ attitudes toward medical errors, manner of disclosure, and the approach to colleagues’ errors (30).

In Iran, there have been few studies on medical errors and design of an appropriate questionnaire in this regard, and we found none that focused specifically on the validity and reliability of such a questionnaire. Moreover, all existing literature overlaps with certain sections of the present study. Thus, our attempt was to design a valid and reliable questionnaire to measure medical error disclosure and related issues. 

In a 2013 study by Tagaddosinejad et al. in Iran, a questionnaire containing 20 questions was used. The questionnaire examined the occurrence of medical errors by physicians and their attitude toward disclosing the types of medical errors to patients, reporting medical errors to authorities and colleagues, prosecution following disclosure of medical errors to patients, and the reasons for non-disclosure of errors by physicians. In this study, medical errors were categorized as major, minor and near-miss errors. The items related to the rate of occurrence of medical errors in our questionnaire are similar to those of Tagaddosinejad et al., although we only investigated the six previous months. Our study also examined physicians’ attitudes toward the manner of medical error disclosure and their colleagues’ errors, which have not been addressed in the study by Tagaddosinejad et al. (31).

Our findings are in line with some studies that obtained similar ICC and Cronbach’s alpha values. In a 2011 study about medical errors conducted on 80 Iranian general practitioners in the city of Zabol, at the test-retest stage, the correlation coefficient was 0.80 (32). This questionnaire consisted of 19 questions, which evaluated the rates, types and preventive factors of medical errors, physicians’ attitudes toward them, and the factors involved in their non-disclosure. In our study, aside from medical error preventive factors, other issues were evaluated, and along with the manner of medical error disclosure, the approach of physicians in dealing with their colleagues’ errors was also taken into account. 

In a pilot study about medical error disclosure on 273 medical residents and interns in Kerman University of Medical Sciences, the reliability of the questionnaire calculated by Cronbach’s alpha was 0.80. This study examined the medical error experience, the type of error (major, minor, or near-miss), the reasons for concern about medical error disclosure, and the attitude of the participants toward medical errors (33). Our study examined all of the above issues more extensively and had other aspects, as discussed earlier.

Another study on the subject was conducted by Ghalandarpoorattar et al. on 53 faculty members and residents of hospitals affiliated to Tehran University of Medical Sciences. It investigated the physicians’ attitudes toward two types of major and minor medical errors, the level of willingness to disclose errors, effects and barriers pertaining to error disclosure, and participants’ practices regarding their own errors. Despite similarities in the general topics, in the study by Ghalandarpoorattar et al., the questions in each domain differed from those in our study, and they reported no reliability testing (34).

Thus, the various questionnaires used in the above-mentioned studies only partially investigated our intended points of the issue, which justifies our construction of the present questionnaire. However, we do not claim that our instrument is a comprehensive questionnaire covering all aspects of medical errors, although it can be useful in investigating the status of medical errors, particularly ethical considerations. 

Medical error recording and reporting systems, whether in paper or electronic form, and with either optional or compulsory approaches to document, follow up, and manage errors, are supposed to prevent their reoccurrence.

Although the guideline for management of this adverse event has been communicated to medical universities, there is currently no system for recording or reporting errors in Iran, so the incidence of medical errors is relatively unknown. Therefore, this questionnaire may not only be helpful in measuring the incidence of medical errors, it can also be used to investigate other factors related to medical errors, including physicians’ attitudes toward dealing with errors and their disclosure, and the manner in which they are disclosed. None of the available questionnaires could simultaneously examine these issues, and to use related questionnaires in international studies, localization and psychometrics of the instruments needed to be performed.

In conclusion, awareness of physicians’ attitudes toward medical errors through identification of the strengths and weaknesses will assist policy makers and healthcare managers in planning to enhance physicians’ professional skills to improve relationships. It can also improve the quality of health care provision and support patient autonomy, and develop and maintain trust in the patient-physician relationship. Thus, a valid and reliable questionnaire is necessary to explore the status of medical errors by physicians, their approach in dealing with such errors, and their attitude toward medical error disclosure.

## Notes:

**Citation to this article:**

Mohammadi M, Larijani B, Tabatabaei SM, Nedjat S, Yunesian M, Nayeri FS.

A study of the validity and reliability of the questionnaire entitled “physicians' approach to and disclosure of medical errors and the related ethical issues”. J Med Ethics Hist. 2019; 12: 2.
